# Tissue-Nonspecific Alkaline Phosphatase (TNAP) as the Enzyme Involved in the Degradation of Nucleotide Analogues in the Ligand Docking and Molecular Dynamics Approaches

**DOI:** 10.3390/biom11081104

**Published:** 2021-07-27

**Authors:** Rafal Madaj, Bartlomiej Gostynski, Roza Pawlowska, Arkadiusz Chworos

**Affiliations:** Division of Bioorganic Chemistry Centre of Molecular and Macromolecular Studies, Polish Academy of Sciences, Sienkiewicza, 112, 90-363 Lodz, Poland; rmadaj@cbmm.lodz.pl (R.M.); bgostyns@cbmm.lodz.pl (B.G.)

**Keywords:** TNAP, alkaline phosphatase, nucleotide analogues, bisphosphonate derivatives, molecular docking, molecular dynamics, thermodynamic integration, multiple sequence alignment

## Abstract

Tissue-nonspecific alkaline phosphatase (TNAP) is known to be involved in the degradation of extracellular ATP via the hydrolysis of pyrophosphate (PPi). We investigated, using three different computational methods, namely molecular docking, thermodynamic integration (TI) and conventional molecular dynamics (MD), whether TNAP may also be involved in the utilization of β,γ-modified ATP analogues. For that, we analyzed the interaction of bisphosphonates with this enzyme and evaluated the obtained structures using in silico studies. Complexes formed between pyrophosphate, hypophosphate, imidodiphosphate, methylenediphosphonic acid monothiopyrophosphate, alendronate, pamidronate and zoledronate with TNAP were generated and analyzed based on ligand docking, molecular dynamics and thermodynamic integration. The obtained results indicate that all selected ligands show high affinity toward this enzyme. The forming complexes are stabilized through hydrogen bonds, electrostatic interactions and van der Waals forces. Short- and middle-term molecular dynamics simulations yielded very similar affinity results and confirmed the stability of the protein and its complexes. The results suggest that certain effectors may have a significant impact on the enzyme, changing its properties.

## 1. Introduction

Human tissue-nonspecific alkaline phosphatase (h-TNAP) is a homodimeric, membrane-located enzyme that may exist in exocytic soluble form in extracellular space, albeit it has been also identified in mitochondria [1]. The presence of this enzyme has been detected in numerous tissues, including the kidney, liver and bones, as well as the central nervous system [2,3]. Numerous studies indicate its involvement in calcification disorders [4], neuronal development, synaptic function [5], interactions within the brain–immune axis [3,6], thermogenesis [1] and cancer.

TNAP is primarily known to hydrolyze pyrophosphate (PPi) to inorganic phosphate (Pi) and therefore regulate the extracellular PPi/Pi ratio. Through participation in the degradation of extracellular nucleotides, this enzyme is involved in the regulation of purinergic signaling [5]. The activity of nucleotides and their analogues in the extracellular space may be regulated by the presence and activity of the ectonucleotidases. Nucleotide hydrolysis causes an increased level of degradation products, such as nucleosides and pyrophosphate, as well as their analogues in the case of modified nucleotides. Such microenvironmental changes could have serious biological consequences [5,7]. Therefore, during the design of new nucleotides analogues, it is important to ascertain their possible interactions with potential targets.

Some of the natural and synthetic TNAP substrates have already been discovered. Beside the pyrophosphate, identified TNAP substrates are also pyridoxal-5′-phosphate (active form of vitamin B6), phosphocreatine and adenosine phosphates [1,5]. Apart from the possible intracellular substrates, due to the involvement of TNAP in the development of various pathological states, new effective TNAP inhibitors are constantly being designed and synthesized [8,9,10,11,12,13].

In the current work, we analyze the possible interaction of TNAP with pyrophosphate analogues containing sulfur, nitrogen, carbon atoms or hypophosphoric moiety instead of bridging oxygen atoms. In order to verify the hypothesis that atom bridging phosphates are fundamental for forming a complex with TNAP, we decided to perform a thorough in silico study of pyrophosphate and its selected derivatives: monothiopyrophosphate, imidodiphosphate, methylenediphosphonic acid and hypophosphate and three carbon bridge-based drugs, alendronic, pamidronic and zoledronic acids. The methods that were applied in order to address the hypothesis included homology modeling, molecular docking and molecular dynamics simulations, both conventional and alchemical ones. Homology modeling was applied in order to acquire the spatial structure of the protein, as it was not available in the PDB database, and it was based on the known structure of another enzyme, placental alkaline phosphatase. Following homology modeling, molecular docking of the selected phosphates was performed in order to investigate the protein’s possible binding sites and obtain the initial binding affinities for each ligand. The structures obtained through molecular docking are the starting point for alchemical (docked pyrophosphate as the starting structure for TI mutation into other ligands) and canonical molecular dynamics refining simulations (docked ligands as separate starting structures for MM-GBSA). The results of these refined simulations were subsequently processed in order to obtain ligand–enzyme free energy binding. Among the variety of thermodynamic potentials, arguably the most important is the free energy *G* changes, which carry the information about whether the reaction considered is a spontaneous process or not. Of important epistemological value are not only the simple changes in the free energy for a given system during the course of a certain reaction (Δ*G*) but also changes in the free energy between different systems provided the considered reaction remains the same (ΔΔ*G*). Knowledge of the ΔΔ*G* value allows differentiating, e.g., between ligands of different binding strength to the active center of an enzyme. This binding capability is of crucial interest here. There are a number of methods for obtaining (Δ)Δ*G* values in a particular system and the reaction of interest, e.g., perturbation or nonequilibrium methods and their more detailed descriptions are contained in the abundant literature, see for example [14]. In this work, however, among other methods, we decided to implement thermodynamic integration (TI). TI—first proposed by Kirkwood in the 1930s [15] and despite having various difficulties [16,17]—remains to this day one of the most robust and widely used methods for the accurate calculation of differences in the free energy value of a system. The main assumption underlying the method is the possibility of a continuous transition between the free energy surface (FES) basin of one well-defined system (state A) to another one of a different well-defined system (state B). Assuming that the Hamiltonian of the system in states A and B can be described by *H*_A_(*p,q,λ*) and *H*_B_(*p,q,λ*), respectively, where the variables are the atomic linear momenta *p* and positions *q*, and *λ* is an artificial, the coupling parameter added in order to enable integration along the particular path of interest *H*_A_(*λ* = 0) →*H*_B_(*λ* = 1) in the following Equation (1) holds:

(1) $$\Delta G=\int_{\lambda =0}^{\lambda =1}{\left\langle \frac{\partial H\left(p,q,\lambda \right)}{\partial \lambda }\right\rangle }_{\lambda }d\lambda$$

Most importantly, as the free energy *G* is a state function, in order to be able to calculate its difference between two particular states of interest, the path connecting their respective basins does not need to be physically but only computationally realizable and integrable along the limits of integration. Practically, the integration is performed numerically, along a set of several discrete states (“windows”)—each with its own, fractional value of λ. To obtain a sufficiently accurate description of relative ΔΔ*G* changes in our work, we decided to perform a set of such unphysical (so called alchemical) free energy calculations for a system consisting of various organophosphorus ligands bound to the active center of TNAP. Therefore, the aforementioned Δ*G* (Equation (1)) can be understood as a difference of binding ability to the protein binding cavity between various ligands (ΔΔ*G*bind). The differences in relative binding free energy were calculated with TI, assuming that ligands undergo alchemical interchange into one another, i.e., individual (groups of) atoms are computationally interchanged, and λ is the parameter determining the degree of the artificial replacement between states A and B that are understood as containing different ligands in the active center.

An analysis of the formed complexes using one of the end-point free energy methods based on the final state of the system—molecular mechanics generalized Born surface area (MM-GBSA)—was also performed. This method is based on the difference between the thermodynamic potential values of a ligand-bound and ligand-free protein in an implicit solvent environment. In general, it can be described using the following Equation (2):

(2) $$\Delta G_{bind}=G_{complex}-G_{protein}-G_{ligand}=\Delta E_{MM}+\Delta G_{solv}-T\Delta S$$

The algorithm applied for these calculations takes into consideration the change in the internal energy and electrostatic energies (Δ*G*_int_ and Δ*G*_ele_, which create the Δ*G*_MM_ term), solvation energy Δ*G*_sol_ and the conformational entropy of the ligand when binding to the receptor. In addition, the algorithm can also yield the contribution of each residue in the protein of interest to the binding of the ligand that facilitates the influence of the point mutations on binding affinity. Through canonical molecular dynamics simulations, we observe the evolution within the selected time through a numerical solution of Newton’s equation of motion, with forces and potential energies calculated using parameterized force fields. Corrections related both to the solvation enthalpy and to the conformational entropic contributions are calculated by treating the solvent as a continuous medium—this later becomes relevant in light of our results (see Molecular Dynamics and MM/GBSA Section).

The aim of this project is to investigate the influence of the chemical modification of the interphosphate atom of the pyrophosphate ligand on binding to TNAP using three completely different techniques ranging from the most rudimentary (molecular docking) through to those of medium sophistication (MM/GBSA) to the most strict ones (TI) and to discuss the possible outcomes of such modification regarding the enzyme activity, as well as assessing the reliability and validity of those approaches in the context of our objectives’ references.

## 2. Materials and Methods

### 2.1. The Structure Preparation

**Figure 1:**
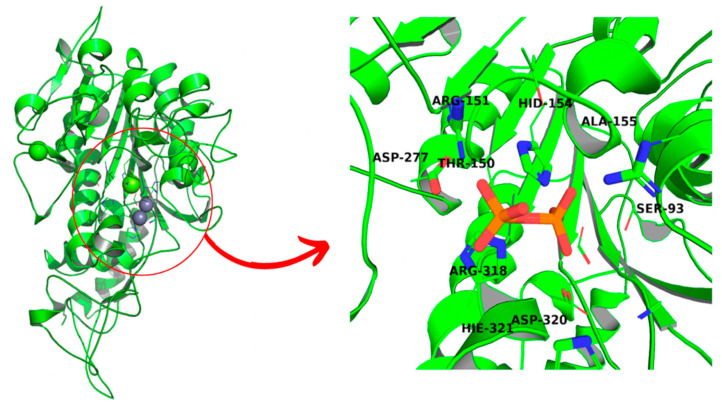
Spatial structure of TNAP, based on PLAP analogue, with enlarged binding cavity. Natural substrate, pyrophosphate, is bound to an enzyme after molecular docking.

The structure of the protein was built using homology modeling. HHpred toolkit [18] was used for finding the homologue of the enzyme. As the closest relative, placental alkaline phosphatase was proposed by the algorithm (PDB entry: 3MK1), with an identity of 57% yielded by T-Coffee MSA analysis [19]. Subsequently, the spatial structure of TNAP was built using Modeller software [20], followed by its minimization using UCSF Chimera software [21] and structure validation via SAVES6.0 and ProSA server functionalities [22,23,24] (Figure 1).

The structures of alendronic (CID: 2088), pamidronic (CID: 4674) and zoledronic (CID: 68470), as well as pyrophosphoric acid (CID: 4995), were downloaded from the PubChem database. The pyrophosphoric acid structure was used as a template for building hypophosphoric, methylenediphosphonic, monothiopyrophosphoric and imidobisphosphoric acids. Molecules were subsequently minimized using the general amber force field (GAFF) in the Avogadro 2 suite.

Molecular docking consisted of the following steps: (1) searching the space for a potential binding site, including the whole protein structure, and (2) thorough molecular docking with selected flexible side chains within 5 Å of the docked ligand. The protein was converted using the pdb2pqr toolkit [25] in order to protonate the structure accordingly to a pH of 8.0. Ligands were loaded as mol2 files into AutoDockTools, where they had partial charges assigned and converted into pdbqt files.

### 2.2. Molecular Docking

The docking of molecules was performed using the AutoDockVina module [26]. The docking grid, in search of a binding cavity, was set to X = 44.7, Y = 20.8 and Z = 10.8 with sizes 51.8, 67.0 and 64.0, respectively. After identification of the binding cavity, the grid for the flexible docking study was set to X = 39.9, Y = 14.8 and Z = 4.1 with 23.1, 21.5 and 21.7, respectively. Side chains within 5 Å from the center of the grid were marked as flexible. The number of binding modes was set to 5000, the exhaustiveness of the search set to maximum (100) and energy range to 10 kcal/mol, and, for calculation, the 14 CPU cores were used.

### 2.3. Conventional Molecular Dynamics and Postprocessing

In each case, the protocol described by [27] was followed for the molecular dynamics simulation. However, it was modified by adding three additional 100 ns simulations of the apo-protein and each protein–ligand complex to validate the results of the shorter simulations. For the selected drugs, the analysis was limited to only 100 ns simulations. All calculations were carried out using the AMBER18 suite [28]. The validation of the simulations, performed using CPPTRAJ [29], was carried out through the radius of gyration (Rg), the root mean square deviation (RMSD) of the protein backbone and ligand and the solvent accessible surface area (SASA) of the ligand throughout the simulation. The analysis was further extended with DSSP [23,30] for the identification of residues contributing to the secondary structure. In order to calculate the relative binding affinity of the selected ligands, molecular mechanics generalized Born surface area (MM/GBSA) calculations [31] were performed, based on each 10th frame of the merged production trajectories, with the generalized born model (igb) set to 5 and a salt concentration of 0.15 M. For shorter simulations, we also applied pairwise decomposition to investigate the residue contribution to the complex formation.

### 2.4. Thermodynamic Integration

The thermodynamic integration (TI) technique, in order to estimate ΔΔ*G* differences between the natural substrate and other phosphate derivatives, was applied. The preparation of simulations consisted of a proper selection of softcore atoms (Table 1), initial minimization and the heating and equilibration of both ligand and complex systems, followed by further preparation of input topologies and coordinates. Minimization consisted of 10,000 steps, including 5000 conjugate gradient ones, with restraints put on the hydrogen atoms and the solvent of 5 Å^2^, followed by 500 ps heating from 50 K to 300 K and equilibrated for 20 ns, with timestep 1 fs.

Free energy simulations were based on the transformation of the molecules using a set of 12 windows, set exponentially from 0 to 1, as suggested by [32] and the calculation of the Van der Waals forces contribution. Each window consisted of a 5 ns production simulation, during which the algorithm calculated the binding energy differences. This part differs from the previous configuration by removing SHAKE between bonds containing one common and one unique atom, resulting in reducing the timestep to 1 fs.

### 2.5. Calculation Platform

Initial calculations were performed using the Intel^®^ CoreTM i9-9900KF CPU @ 3.60 GHz × 16 with 32 GB @ 2666 MHz with GeForce RTX 2070 SUPER/PCIe/SSE2 (CBMM PAS, Lodz, Poland) on the Ubuntu 20.04 Focal Fossa and subsequently using the PL-GRID infrastructure (Polish Grid Infrastructure PL-Grid, Poland). 

## 3. Results

### 3.1. Structure Validation

The homologue of TNAP, build based on PLAP analogue (Figure 1), passed the tests with an overall quality factor of 78.91 for the ERRAT analysis and an overall Z-score of −8.29. The Ramachandran plot suggests that while the majority of residues are placed in favored regions, some are not, with few amino acids present in disallowed regions. However, such a situation is hard to evade in the case of modeling that is based on a homologue with just 57% identity (Figures S1–S3 in Supplementary Information).

### 3.2. Molecular Dynamics Simulations Validation

**Figure 2:**
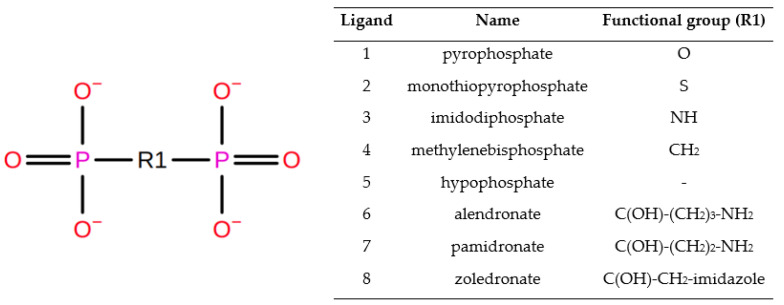
Series of ligands with selective modification at the phosphates connected to O-atom position.

The thermodynamic integration analysis was validated through the investigation of changes in density in time during the equilibration phase (Figures S4–S10), which is the starting point for production simulations. In case of canonical molecular dynamics, each run was subjected to an extended analysis in order to check system stability. Radius of gyration plots (Figures S11 and S12) indicate high stability, about 23 Å, with low fluctuations up to 0.5 Å, with one exception for alendronate, up to 1 Å. Backbone RMSD indicates stability of the structures, regardless of the ligand bound, with an average of 3 Å, but with notable fluctuations (about 0.8 Å, Figures S13 and S14). Worth mentioning is an influence on the average RMSD of the terminal, flexible α-helix formed by residues 1–30 (Figure S19). There is a discrepancy between the RMSD profile of the homologue presented in this study and by [13,33]; however, it is worth mentioning that in each case the models generated differed from one another to some degree. Among the selected ligands: monothiopyrophosphate, imidodiphosphate, methylenediphosphonic acid and hypophosphate and the three carbon bridge-based drugs, alendronic, pamidronic and zoledronic acids (Figure 2), the most stable and tightly bound structures are imidodiphosphate and monothiopyrophosphate with an RMSD of about 0.2 Å (obtained during short-term runs, Figure S17) and about 1 Å (obtained in long-term runs, Figure S18). Nevertheless, these two structures practically did not fluctuate significantly during all the calculations, and this is also observable in the solvent accessible surface area analysis, which yields about 150 Å^2^ of the area accessible for the solvent in each case. However, these time long-term simulations yielded more stable results. The lowest value was obtained for pamidronate, about 50 Å^2^ (Figures S15 and S16); the structure also shows that the ligand is positioned deep inside the binding cavity. The ligand RMSD profile also differs from previous studies [13,33]. However, in the aforementioned publications, triazole, pyrazole and thiazole derivatives were investigated. They are also notably larger and carry no formal charge.

### 3.3. Molecular Docking and Dynamic’s Results

The obtained results suggest that the binding affinities of phosphate derivatives are similar in the case of very closely related compounds such as pyrophosphate and its derivatives; however, sole molecular docking may not be enough to be accurate. In contrast, molecular dynamics simulations can exclude such problems [34]. Table 1 shows a summary of all obtained binding affinities using each technique investigated in the present study.

**Figure 3:**
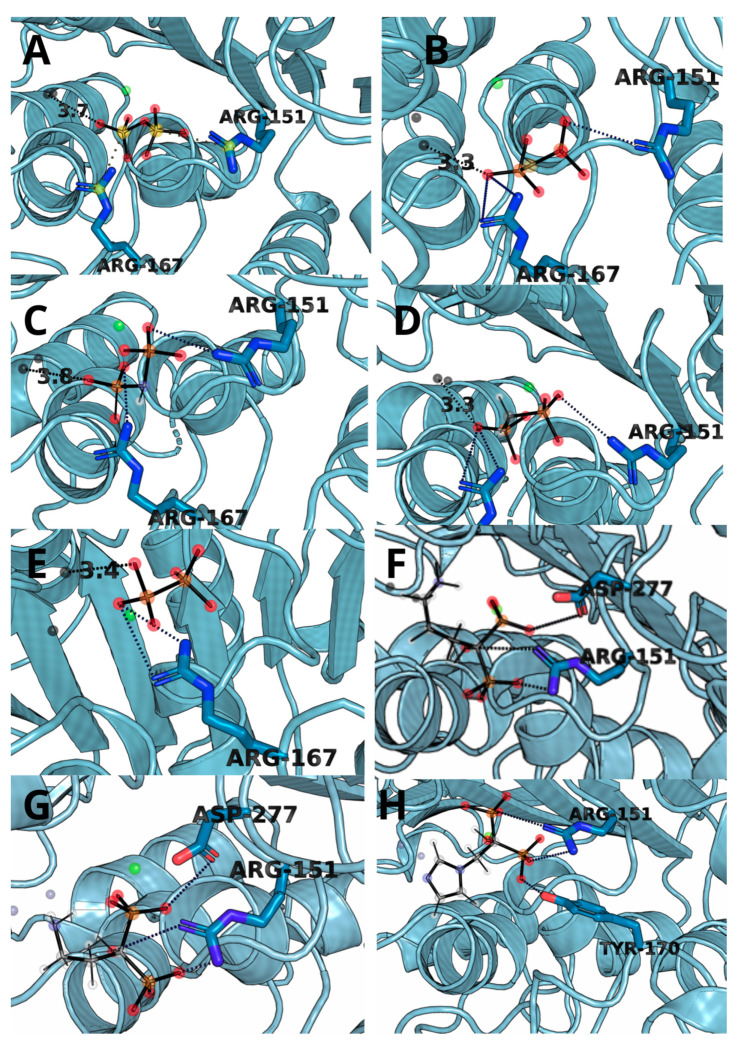
The structure snapshots for TNAP active site with ligands: (**A**) pyrophosphate, (**B**) monothiopyrophosphate, (**C**) imidodiphosphate, (**D**) methylenediphosphonic acid, (**E**) hypophosphate, (**F**) alendronate, (**G**) pamidronate and (**H**) zoledronate.

The complexes obtained through molecular docking, being also starting structures for further molecular dynamics simulations, show very high similarity of not only Δ*G*s but also with ligand placement within the binding cavity. The most important factors of the complex formation were Arg151 and Arg167, capable of forming strong electrostatic interactions and hydrogen bonds and Zn^2+^ cation, which can interact with negatively charged phosphate groups (Figure 3). PLIP algorithm detected multiple interactions between two arginine residues and a ligand in all cases except for hypophosphate. In the case of bisphosphonate, the ligands were complexed by one of the zinc ions present within 4 Å.

### 3.4. Thermodynamic Integration

To obtain a sufficiently accurate description of relative ΔΔ*G* changes in our work, we decided to perform a set of alchemical free energy calculations for a system consisting of various organophosphorus ligands bound to the TNAP active center. With this approach, the aforementioned Δ*G* from Equation (1) can be applied as a difference of binding ability to the protein binding cavity between various ligands (ΔΔ*G*bind). The pyrophosphate from molecular docking was adopted as the starting point. Thermodynamic integration indicates significant influence of the modification of the bridging atom on free energy and the refined results obtained through molecular docking. Softcore atoms being interchanged into one another are presented in Table 2.

**Figure 4:**
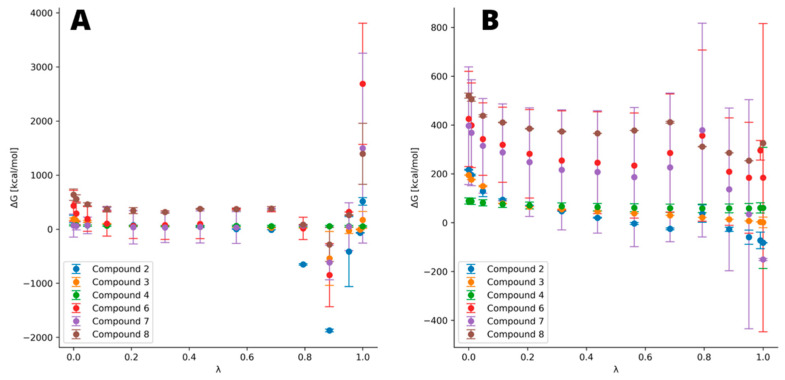
Free energy representation obtained at every λ. (**A**) Ligands bound to the complex and (**B**) free ligands in water box. The higher the Δ*G* nearby λ of 0.8 and the steeper the slope of curve, the higher the difference in total ΔΔ*G*. Notable deviations are observed for drugs (compounds **6**–**8**), which indicate their lesser stability but is also expected due to their significantly bigger structure.

The most notable difference can be observed in the case of thiophosphate derivatives, which change significantly in terms of atom radius, electronegativity and electron number. The results suggest that all derivatives bind stronger to TNAP than the natural substrate, pyrophosphate, whereas monothiopyrophosphate binds significantly stronger to the enzyme than all other ligands, including the drugs (compounds **6**–**8**). Another significant, albeit much smaller, difference between the pyrophosphate and its counterpart is found for imidodiphosphate, yielding ΔΔ*G* of −47.8 kcal/mol. An interesting result was obtained for the methylene derivative, as it suggests that there is almost no difference in ΔΔ*G* between this derivative and the pyrophosphate. However, the investigated drugs (compounds **6**–**8**), based on carbon with hydroxyl group and another depending on the drug, bind notably better than the methylene derivative. Zoledronic acid yielded ΔΔ*G* slightly lower than imidodiphosphate, while alendronic and pamidronic acids give ΔΔ*G* between the one for imidodiphosphate and monothiopyrophosphate. The integration curve in Figure 4 reflects the differences between each ligand. In the case of TI, we ran calculations for estimating the ΔΔ*G* difference between pyro- and hypo-phosphate (data not shown); however, it was impossible to obtain meaningful results. This is due to the limitations in the computational algorithm to bind two existent phosphate groups while removing the bridging atom. Therefore, it was necessary not only to set the oxygen atom as being substituted but also the whole second phosphate group, as shown in Table 2. Due to this unwanted but necessary modification in the procedure, the system was not stable during the calculations and crashed at λ close to both limits and yielded physically impossible results (ΔΔ*G* of about 4500 kcal). Due to this impediment, we were forced to resort to another method in order to assess the binding ability of hypophosphate.

### 3.5. Molecular Dynamics and MM/GBSA

Interactions investigated by TI were thus subsequently repeated and extended through computationally less demanding MM/GBSA with pairwise decomposition studies—we decided not only to calculate the lacking hypophosphate binding affinity but to run computations for all the other ligands as well in order to assess in this way the reliability of this common, relatively low-level method in comparison to the much more thermodynamically and statistically strict TI in the case of our system. Postprocessing applied to the obtained trajectories indicates the important role of metal ions (especially Zn^+2^ and Mg^+2^) and arginine residues in complex stabilization, which is in overall agreement with the literature [5] describing the possible mechanism for these types of enzymes [24,35]. However, while in the case of pyro, hypo and all methylene derivatives Mg^+2^ is less influential, in the case of imido and thio analogues, its role is as important as zinc ions (res. 482, Table 3). Worth mentioning is the fact that the amino acid residue contribution to substrate binding was the highest for pyrophosphate.

**Figure 5:**
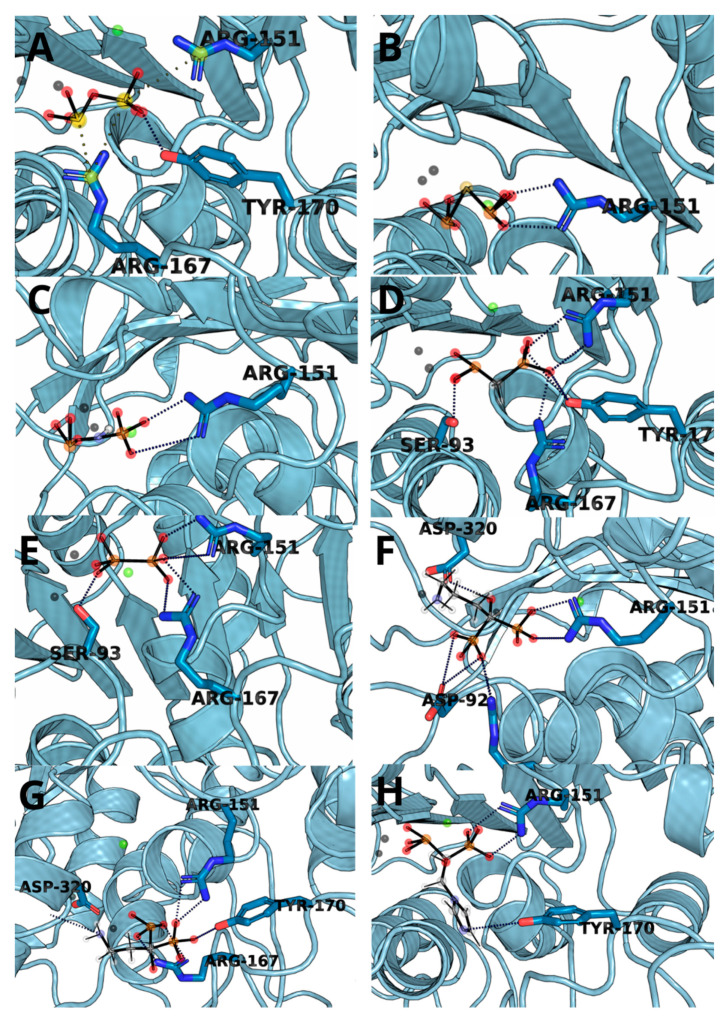
Representative spatial orientation of (**A**) pyrophosphate, (**B**) monothiopyrophosphate, (**C**) imidodiphosphate, (**D**) methylenediphosphonic acid, (**E**) hypophosphate, (**F**) alendronate, (**G**) pamidronate and (**H**) zoledronate from the molecular dynamics simulation. MD investigation indicates significant influence of metal ions (zinc—black sphere, magnesium—green sphere) on ligand binding, as they are close (apart from alendronate) to deprotonated moieties and yield low contribution energies.

**Figure 6:**
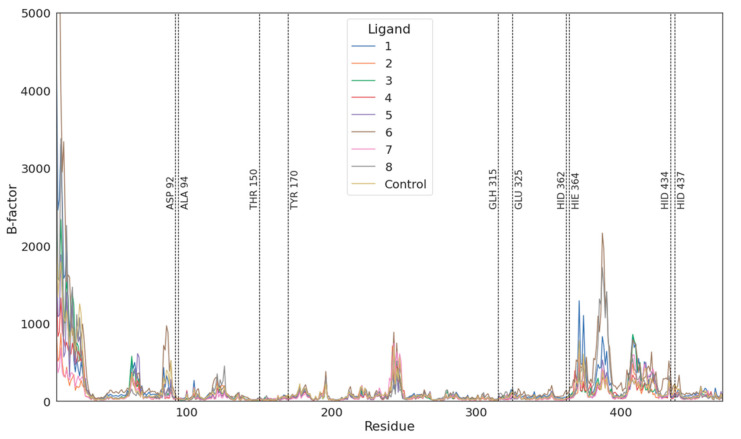
Fluctuations of residues 20–450 during simulations. Vertical lines indicate residues in the binding vicinity. There are major shifts nearby residues 250, 320 and 420, which are regions voiding any secondary structures.

The amino acids involved in ligand binding are presented in Figure 5, as a representative from clustering the trajectories. In each case, negatively charged atoms form ionic interactions with metal ions, Zn cation or, in the case of imidodiphosphate, with Mg cation. There are also a number of interactions between the ligand and arginines or another residue in their vicinity, mainly being hydrogen bonds and electrostatic interactions. All these interactions resulted in overall negative Δ(Δ)*G* values obtained through both TI and MM/GBSA studies. There is also a strong influence of negatively charged phosphate groups (total charge -4), which when combined with the divalent metal ions in the vicinity (Mg^2+^ and two Zn^2+^) have influence on the energy of binding. The fact that hypophosphate binds weaker than the natural substrate derives straight from the fewer number of possible interactions. As hypophosphate lacks a bridging atom, which in every case but methylenediphosphonic acid is electronegative, the number and strength of potential interactions are lower and so is the hydrophobic effect from the carbon atom from methylenediphosphonate. In most cases, ligands have limited influence on the fluctuation of residues in the active site (Figure 6). The only region that seems to be changed by ligand binding is between 370 and 430, which changed its spatial structure in the case of hypophosphate and alendronate. The origin of this phenomena may derive from the interactions with the effector that is deeply buried in the binding site.

**Figure 7:**
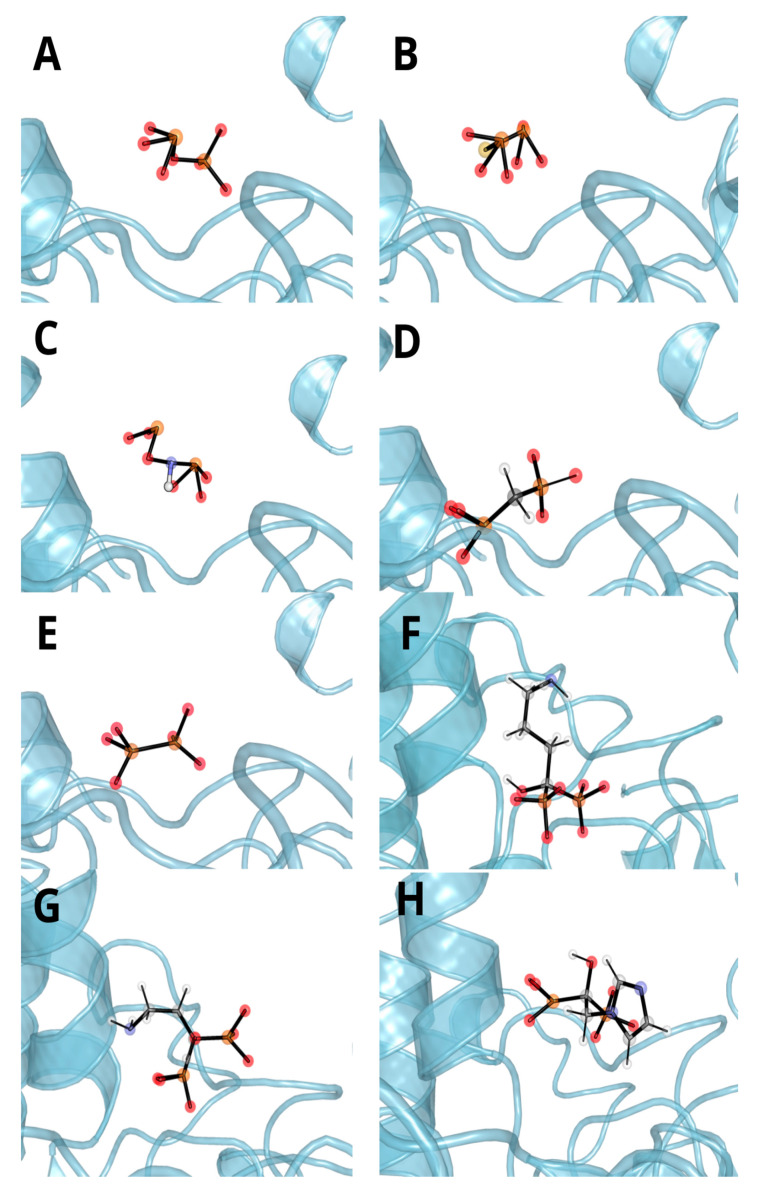
Ligand in the TNAP cavity for (**A**) pyrophosphate, (**B**) monothiopyrophosphate, (**C**) imidodiphosphate, (**D**) methylenediphosphonic acid, (**E**) hypophosphate, (**F**) alendronate, (**G**) pamidronate and (**H**) zoledronate from clustered trajectories.

One may notice that the Δ(Δ)*G* values obtained during this investigation are somewhat different from the results obtained before for other ligands and are also different when comparing TI to MM/GBSA (Table 1). The first discrepancy in energy differences obtained may be justified by the fact that they are relative to the energy of the (different) free reagents forming the particular (different) system(s) of interest. The second, however, points to the fact that the results from MM/GBSA should differ qualitatively from the TI ones. According to the MM/GBSA method, the imido derivative binds stronger to TNAP than the thio derivative (which is the best according to the TI calculations), and, also, the methylene derivative binds worse than the pyrophosphate. The main source of enormous error in this case, we assume, may be twofold: the approximate treatment of the solvent as a continuous medium when calculating ΔG_solv_ correction (Equation (2)) for the MM/GBSA method. Indeed, when inspected, the calculated structures reveal that the imido derivative, when compared to the other ones, is inserted in the TNAP binding cavity in such a way that one of its charged phosphoryl groups protrudes slightly from the cavity (Figure 7). This may lead to the overestimation of energetically favorable solvation effects.

The methylene derivatives are buried down too deeply (indicated by residue fluctuation in Figure 6); thus, the interaction with the solvent may be underestimated. Moreover, the above considerations are in overall agreement with the differences in the solvent accessible surface area (SASA) of the ligands (Figures S15 and S16). 

## 4. Discussion

The obtained results do not give a direct comparison of the MM-GBSA method to TI; however, MM-GBSA seems to be not only a system-dependent—a feature well known in the literature [36,37,38]—but also an initial condition-dependent method, and its outcomes should be taken with increased caution. Nevertheless, it is taken into consideration that the hypophosphate might indeed bind significantly weaker than other derivatives because of the following: (a) The calculations for the hypo derivative do not show such strong deviations in SASA or residue fluctuation as for the imido and methylene derivatives, which may disturb the results’ solidity, and the hypo molecule does not possess any atoms bridging the two phosphoryl groups, which in turn results in decreased interactions with the TNAP active site due to the fewer number of possible interactions. As it lacks bridging atoms, which in every case but methylenebisposphonate carries an atom with available electron pairs, the number and strength of potential interactions (e.g., hydrogen bonds, favorable hyperconjugation effects) should be lowered. (b) The calculations for the hypo derivative do not show such strong deviations in SASA or residue fluctuation as for the imido and methylene derivatives, respectively, which may disturb the results’ solidity; one can thus acknowledge the results suggesting that this derivative is the worst binder to TNAP than any other ligand investigated here. In vitro investigation is needed in order to verify the thermodynamic stability of the complex and the hypo derivative to subsequently undergo hydrolysis.

The obtained results may be useful to predict the degradation rates of active molecules, such as β,γ-modified nucleotides or bisphosphonates in extracellular space. They may also be valuable data for designing new potential TNAP-interacting molecules. Our previous study [27] indicated that the majority of investigated bisphosphonates may interact with both alkaline phosphatases, affecting their function; however, obtained affinities differed significantly.

Since TNAP is involved in the degradation of extracellular nucleotides via the hydrolysis of pyrophosphate, it may be assumed that this protein also participates in the degradation of nucleotide analogues. Among this class of compounds, especially interesting are β,γ-modified nucleotides, such as bisphosphonates [39,40], hypophosphates [41] and ATP derivatives, which may be hydrolyzed by ectonucleotidases with the release of modified pyrophosphate analogues. TNAP is considered as a potential target for anti-calcification drug design [4]. Due to its role in brain microvascular dysfunction and neuroinflammation in late sepsis [3], it seems to also be a good target for such disorders. 

## 5. Conclusions

In this paper, we compare TNAP ligand binding energies obtained from three different computational methods, namely molecular docking, thermodynamic integration (TI) and conventional molecular dynamics (MD). For that, we selected a series of pyrophosphate derivatives: pyrophosphate, hypophosphate, imidodiphosphate, methylenediphosphonic acid, monothiopyrophosphate, alendronate, pamidronate and zoledronate. In silico analysis suggests that almost all the investigated compounds bind to the TNAP, with the highest affinity for imido- and thio-phosphate moieties. The hypo derivative is a weak ligand for TNAP and so are the investigated drugs (alendronate, pamidronate and zoledronate); however, to verify that, an experimental analysis is certainly needed to clarify the issue as to whether or not the molecule is able to actually create a thermodynamically stable complex within the active center. Regardless of these ambiguities, it becomes apparent that the type of group bridging the phosphate moieties is certainly an important factor in the energetics of the forming protein–ligand complex. MM/GBSA turns out to be a less reliable and robust method in the estimation of thermodynamics of such processes in comparison to TI, at least in cases of negatively charged phosphate derivatives neighboring metal cations. Such coincidence causes mild malformations of phosphate groups, causing them to point toward metal ions, thus changing the planarity of the group. The MM/GBSA calculation method contains energy of solvation, which is highly inaccurate in such cases. On the other hand, the application of TI is limited to only a molecule sharing a common scaffold, and even this has limitations such as those presented in this paper—hypophosphate could not be treated like another pyrophosphate derivative, as it voids atom bridging phosphate groups. We conclude that in the case of the high similarity of the investigated compounds, the TI method can be recommended due to its higher accuracy and insensitivity to imperfections associated with force fields causing deformations of ligands. However, for the screening of compounds not sharing a common scaffold, voiding problematic groups, such as deprotonated phosphates, MM/GBSA, due to their speed and feasibility may be the first choice.

## Figures and Tables

**Table 1:** Binding affinities of selected ligands toward TNAP. All Δ*G* and ΔΔ*G* values are in [kcal/mol].

Method	Molecular Docking		Molecular Dynamics		
	Average Free Energy of Binding Δ*G*	Lowest Free Energy of Binding Δ*G*	TI ΔΔ*G* in Relation to Pyrophosphate	Total Relative Δ*G* 10 × 10 ns Analysis	Total Relative Δ*G* 3 × 100 ns Analysis
1	−4.3 ± 1.9	−7.7	−	−306 ± 51	−309 ± 54
2	−4.3 ± 1.9	−7.7	−225.8 ± 31	−478 ± 83	−462 ± 24
3	−4.1 ± 1.8	−7.6	−47.8 ± 36	−626 ±77	−627 ± 101
4	−4.3 ± 1.8	−7.8	−7.2 ± 3	−209 ± 26	−205 ± 24
5	−4.2 ± 1.8	−7.3	−	−149 ± 27	−126 ±26
6	−6.8 ± 0.4	7.6	−112.8 ± 161.3	−	−60 ± 69
7	−6.8 ± 0.4	7.7	−198.1 ± 171.8	−	−164 ± 27
8	−7.0 ± 0.3	7.8	−57.3 ± 5.5	−	−153 ± 20

**Table 2:** Atoms selected as softcores for thermodynamic integration.

Ligand	Ligand Softcore	Pyrophosphate Softcore
2	S	O
3	N1,H1	O
4	C1,H11,H12	O
5	PB,O1B,O2G,O3B	P,O,O1,O2,O3
6	C1,N1,C2,C3,C4,O7,HC21,HC31,HC41,H7,H11,H12,H21,H32,H41	O
7	@C1,N1,C2,C3,O7,HC21,HC31,H7,H21,H32,H11,H12	O
8	@C1,O7,H08,C2,H02,H03,N1,C3,H04,C5,H07,N2,C4,H05	O

**Table 3:** Amino acids with contribution to substrate binding energy lower than −5 kcal/mol.

Ligand	Residue Contribution [kcal/mol]							
	SER93	ARG151	ARG167	HIE321	ZN482	ZN483	MG484	OTHER
1	-	−47.36	−50.30	−9.82	−392.74	−42.87	−24.19	TYR170, HID154
2	-	−53.84	-	−10.08	−331.35	−88.61	−387.55	TYR170, HID154
3	-	−47.70	−10.60	−10.29	−497.56	−64.67	−369.11	ARG318
4	−9.63	−40.03	−29.49	−10.93	−259.25	−23.70	−14.05	TYR170
5	−10.93	−16.40	−31.33	−7.18	−229.53	−28.51	−13.90	HID154, ALA155
6	-	−30.31	−33.04	−8.11	−77.8	−11.9	−10.5	HID154
7	−6.19	−24.76	−37.34	−7.35	−244.62	−17.28	−12.62	TYR170, HID154
8	-	−32.11	−9.21	-	−211.28	−6.86	−10.76	-

## Supplementary Materials

The following are available online at https://www.mdpi.com/article/10.3390/biom11081104/s1, Figure S1: ERRAT analysis of TNAP residues. Only few residues crossed values indicating that their spatial orientation may be incorrect, Figure S2: Verify3D analysis. Average score is in close vicinity to desired value (about 0.2), Figure S3: Ramachandran plot of the protein, Figure S4–S10: System density change in time for compounds 1 and 2, 3, 4, 5, 6, 7, 8 respectively, during equilibration prior to TI, Figure S11: Radius of gyration for protein during 20 ns long simulations repeated 10 times (10 ns truncated as an equilibration period). Non-bound protein used as a control, Figure S12: Radius of gyration for protein during 100 ns simulations repeated 3 times (10 ns truncated as an equilibration period). Non-bound protein used as a control, Figure S13: RMSD of CA atoms of the protein during 20 ns long simulations repeated 10 times (10 ns truncated as an equilibration period). Non-bound protein used as a control, Figure S14: RMSD of CA atoms of the protein during 100 ns simulations repeated 3 times (10 ns truncated as an equilibration period). Non-bound protein used as a control, Figure S15: Solvent-accessible surface area of ligands during 20 ns long simulations repeated 10 times (10 ns truncated as an equilibration period), Figure S16: Solvent-accessible surface area of ligands during 100 ns simulations repeated 3 times (10 ns truncated as an equilibration period), Figure S17: Ligand RMSD during 20 ns long simulations repeated 10 times (10 ns truncated as an equilibration period), Figure S18: Ligand RMSD during 100 ns simulations repeated 3 times (10 ns truncated as an equilibration period), Figure S19: Differences in a terminal α-helix and protein core distances between initial structure and clustered trajectory. Distances are between residues: 6 and 58, 16 and 73, 27 and 470.
