# Supplementary material for: Tissue-Nonspecific Alkaline Phosphatase (TNAP) as the Enzyme Involved in the Degradation of Nucleotide Analogues in the Ligand Docking and Molecular Dynamics Approaches
Source: Biomolecules. 2021 Jul 27;11(8):1104. doi: 10.3390/biom11081104 (PMC8391816; doi:10.3390/biom11081104)
Supplement: Supplementary file 1 [file biomolecules-11-01104-s001.zip › biomolecules-1285034-supplementary.pdf]

Supplementary information

Tissue-Nonspecific Alkaline Phosphatase (TNAP) as the Enzyme Involved in the Degradation of Nucleotide Analogues in the Ligand Docking and Molecular Dynamics Approaches

Rafal Madaj, Bartlomiej Gostynski, Roza Pawlowska*, Arkadiusz Chworos*

Division of Bioorganic Chemistry Centre of Molecular and Macromolecular Studies, Polish Academy of Sciences, Sienkiewicza, 112, 90-363 Lodz, Poland; rmadaj@cbmm.lodz.pl (R.M.); bgostyns@cbmm.lodz.pl (B.G.)
*Correspondence: rozapech@cbmm.lodz.pl (R.P.); achworos@cbmm.lodz.pl (A.C.);
Tel.: +48-42-6803215 (R.P.); Tel.: +48-42-6803220 (A.C.)

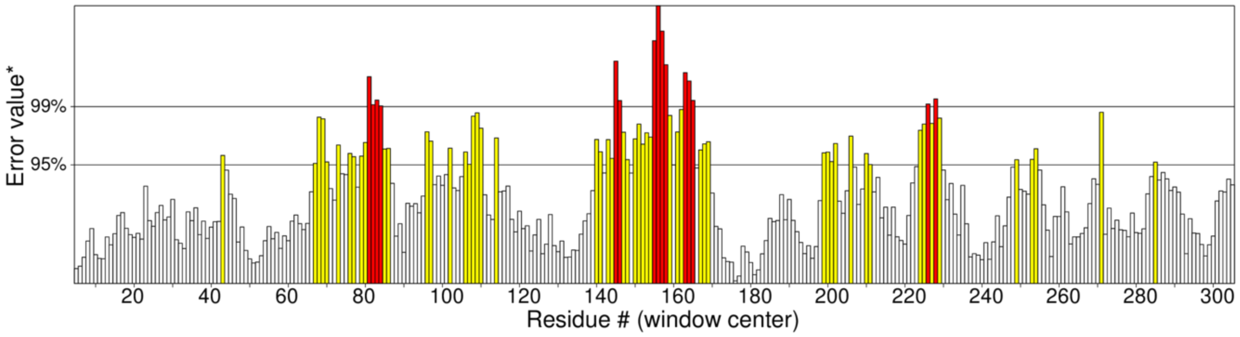

Figure S1. ERRAT analysis of TNAP residues. Only few residues crossed values indicating that their spatial orientation may be incorrect.

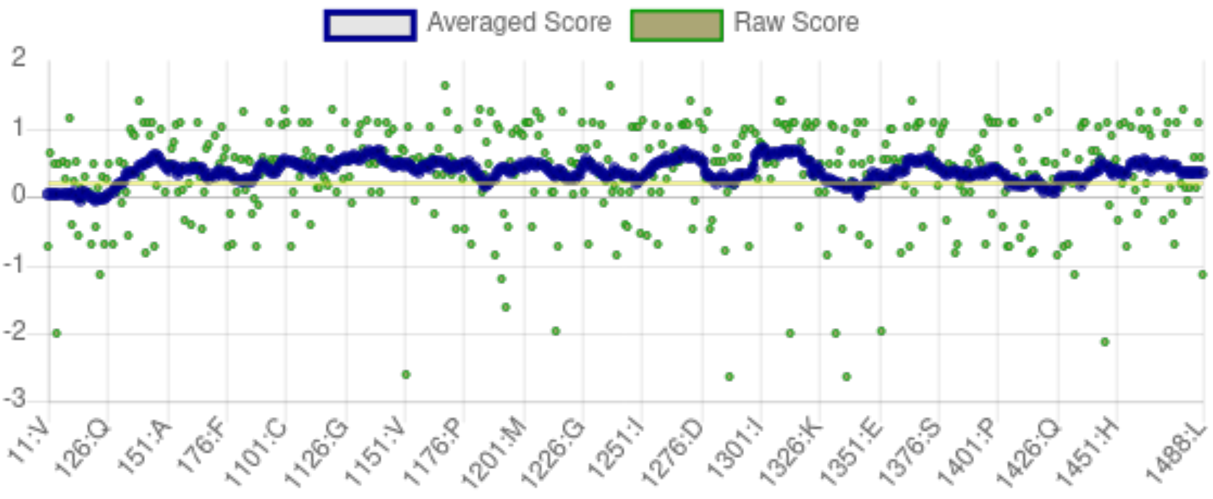

Figure S2. Verify3D analysis. Average score is in close vicinity to desired value (about 0.2)

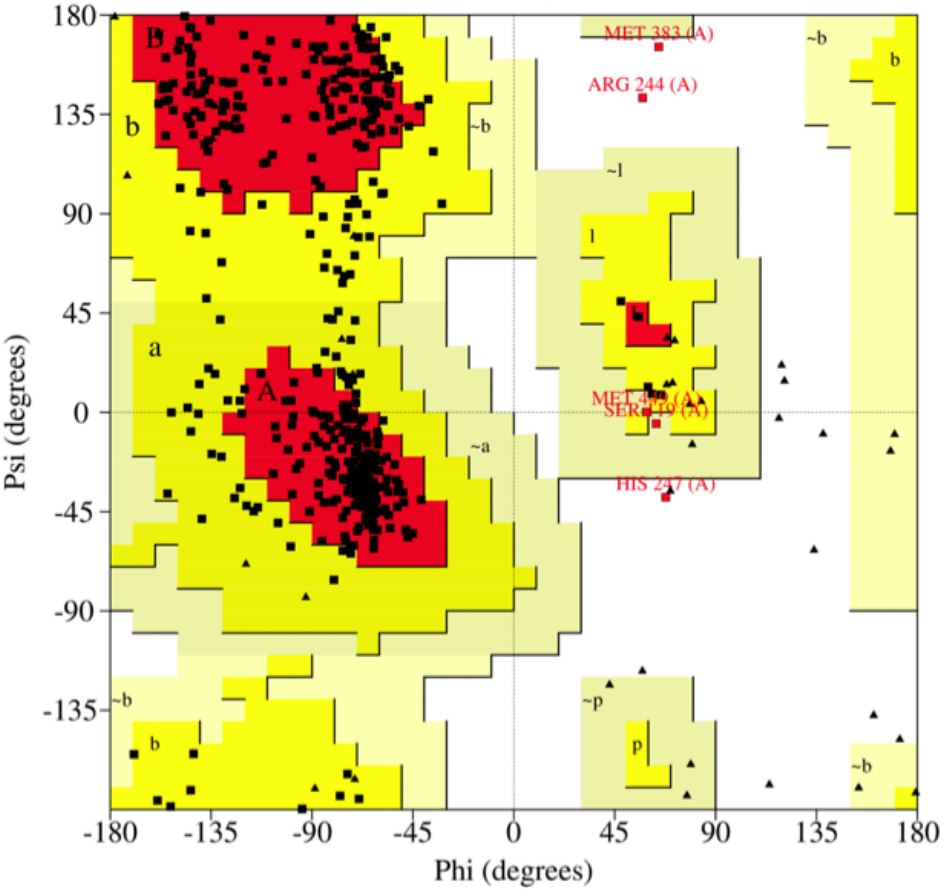

Plot statistics

Residues in most favoured regions [A,B,L]	325	77.2%
Residues in additional allowed regions [a,b,l,p]	91	21.6%
Residues in generously allowed regions [~a,~b,~l,~p]	2	0.5%
Residues in disallowed regions	3	0.7%
	----	------
Number of non-glycine and non-proline residues	421	100.0%
Number of end-residues (excl. Gly and Pro)	425	
Number of glycine residues (shown as triangles)	86	
Number of proline residues	44	

Total number of residues	976	

Based on an analysis of 118 structures of resolution of at least 2.0 Angstroms and R-factor no greater than 20%, a good quality model would be expected to have over 90% in the most favoured regions.

Figure S3. Ramachandran plot of the protein

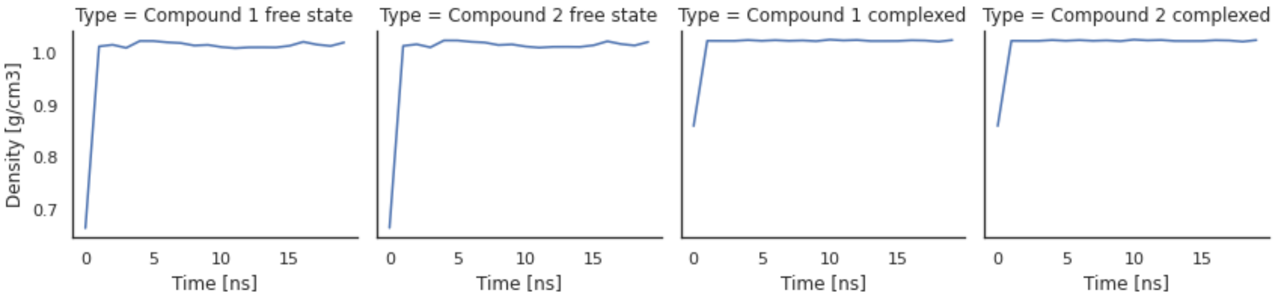

Figure S4. System density change in time for compounds 1 and 2 during equilibration prior to TI

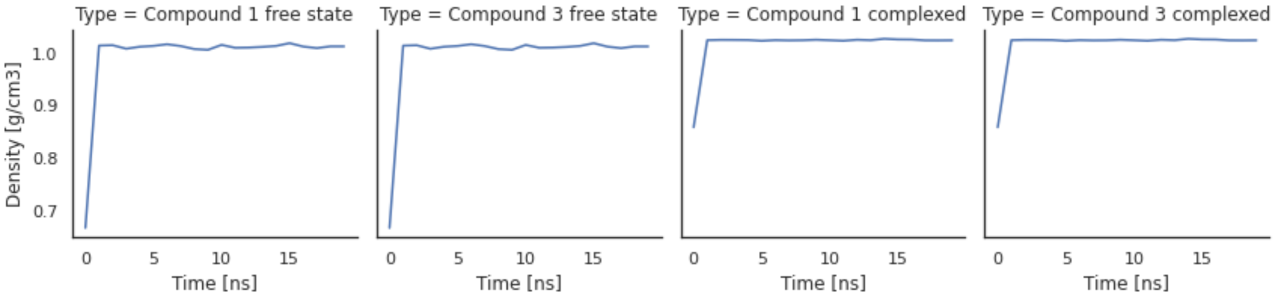

Figure S5. System density change in time for compounds 1 and 3 during equilibration prior to TI

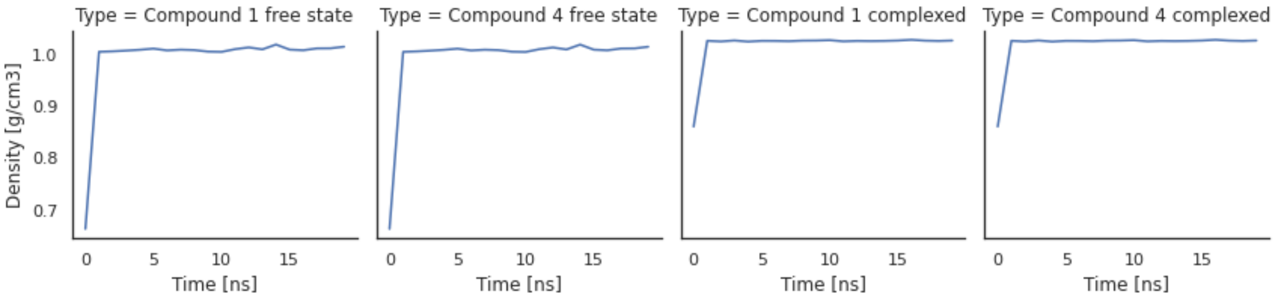

Figure S6. System density change in time for compounds 1 and 4 during equilibration prior to TI

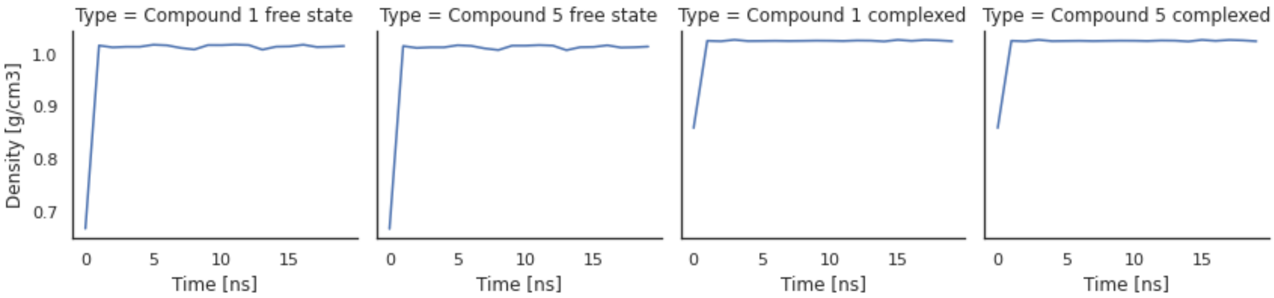

Figure S7. System density change in time for compounds 1 and 5 during equilibration prior to TI

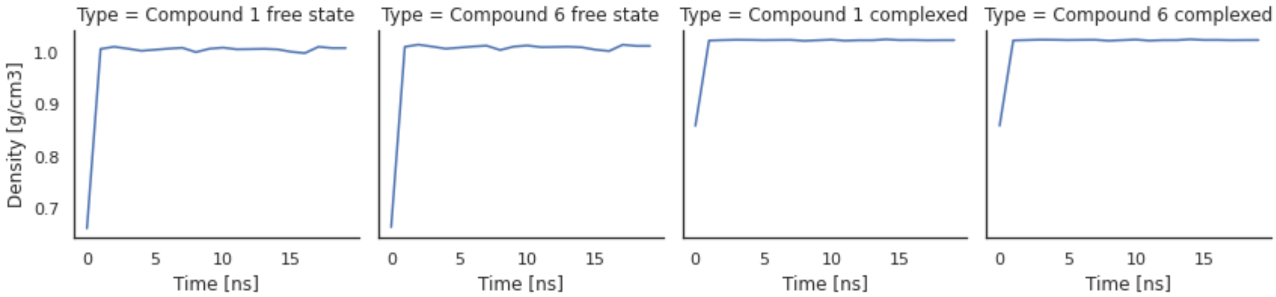

Figure S8. System density change in time for compounds 1 and 6 during equilibration prior to TI

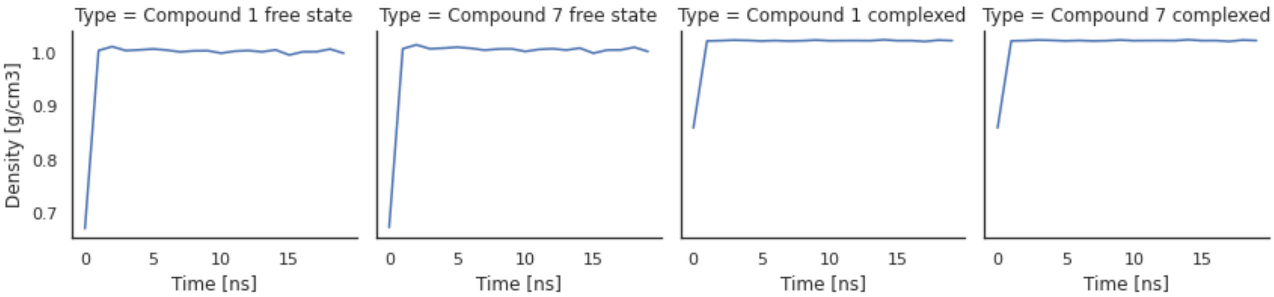

Figure S9. System density change in time for compounds 1 and 7 during equilibration prior to TI

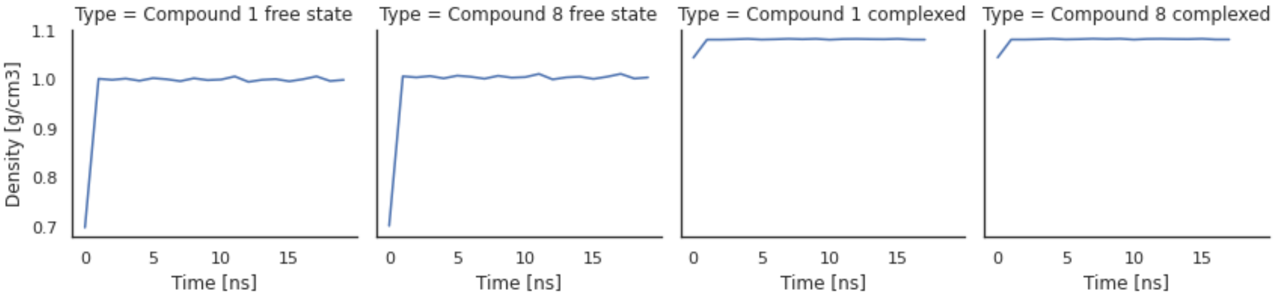

Figure S10. System density change in time for compounds 1 and 8 during equilibration prior to TI

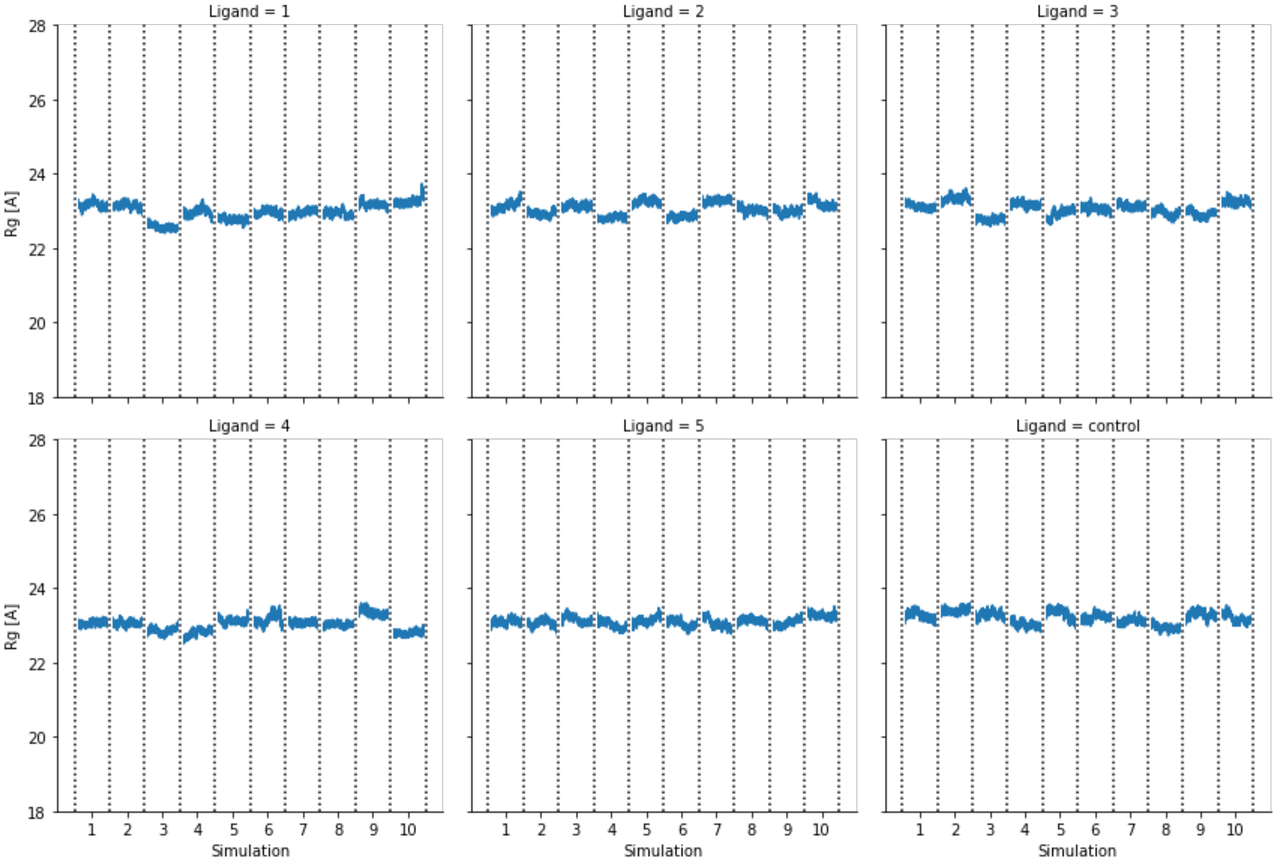

Figure S11. Radius of gyration for protein during 20 ns long simulations repeated 10 times (10 ns truncated as an equilibration period). Non-bound protein used as a control.

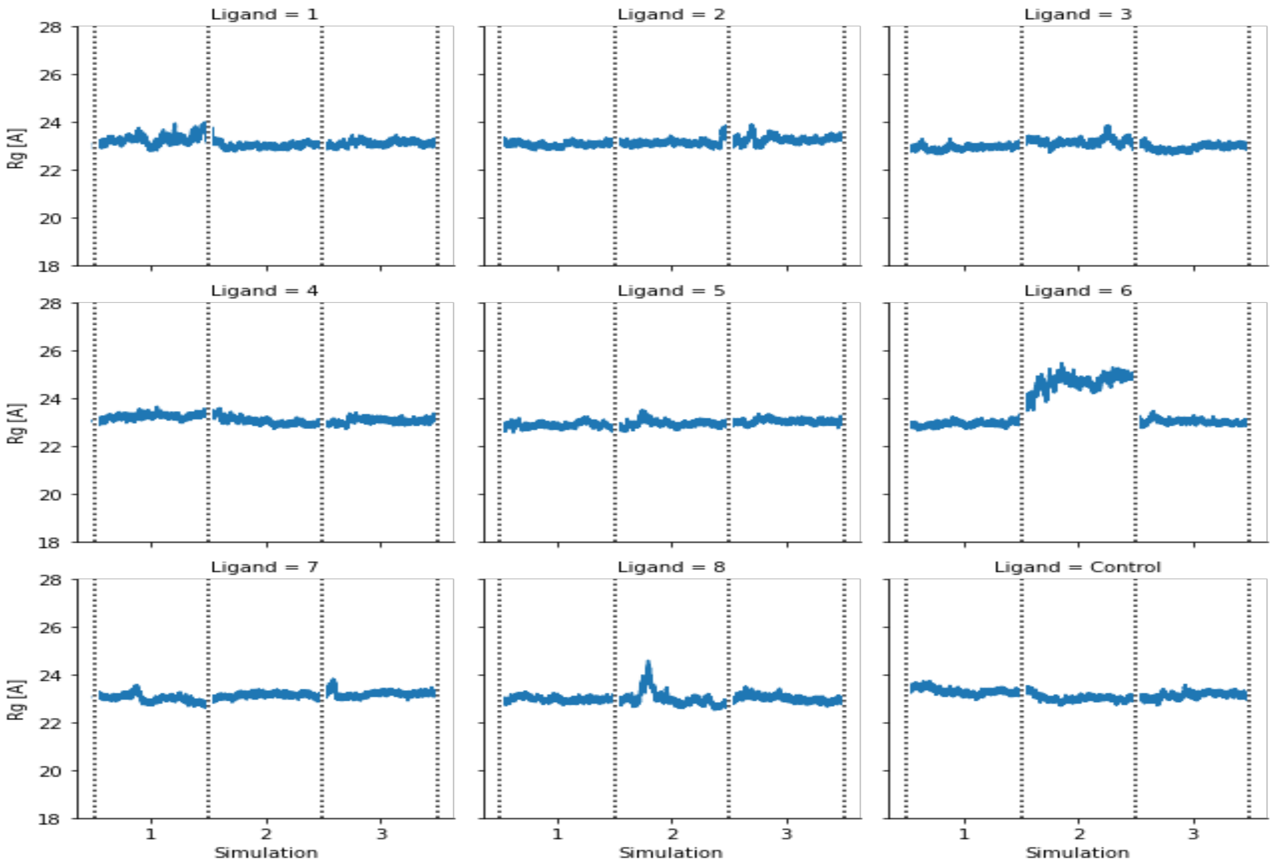

Figure S12. Radius of gyration for protein during 100 ns simulations repeated 3 times (10 ns truncated as an equilibration period). Non-bound protein used as a control.

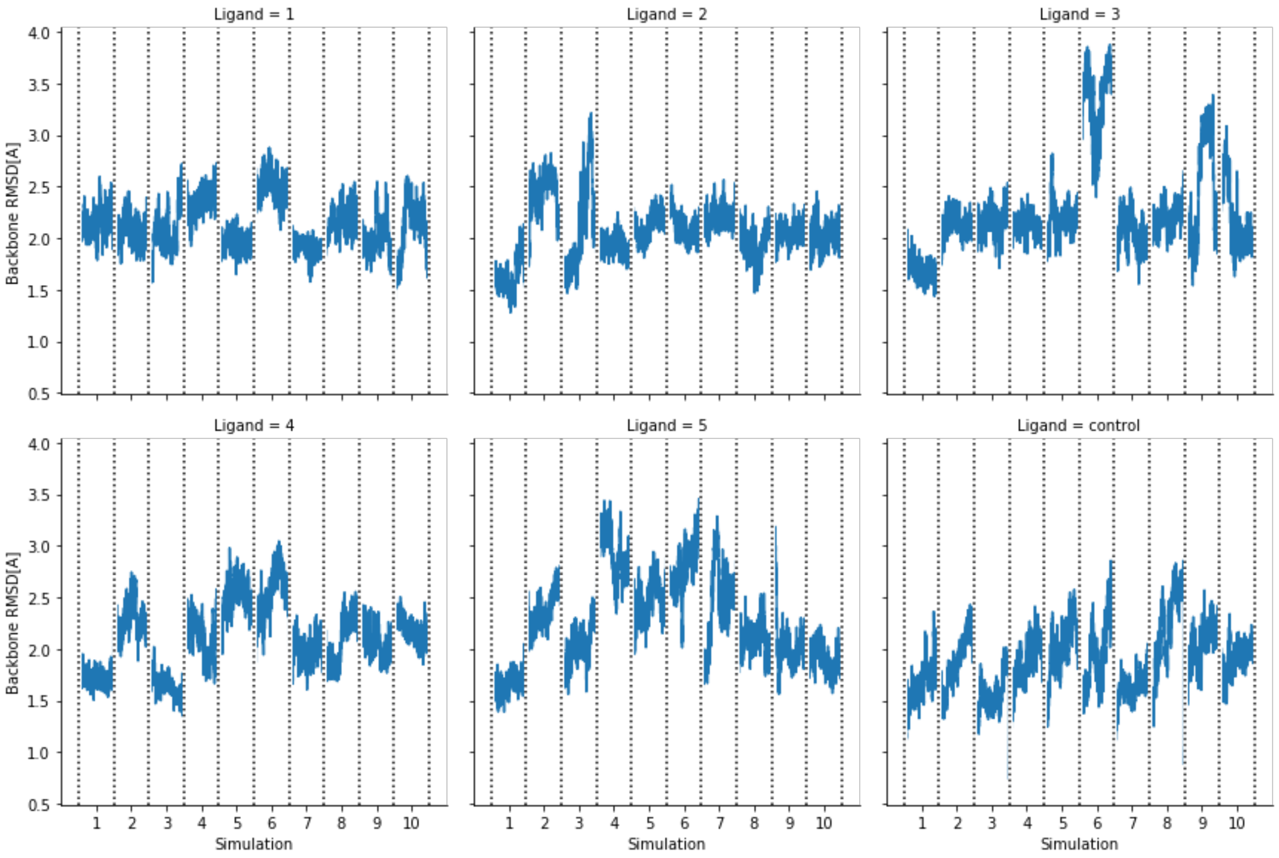

Figure S13. RMSD of CA atoms of the protein during 20 ns long simulations repeated 10 times (10 ns truncated as an equilibration period). Non-bound protein used as a control.

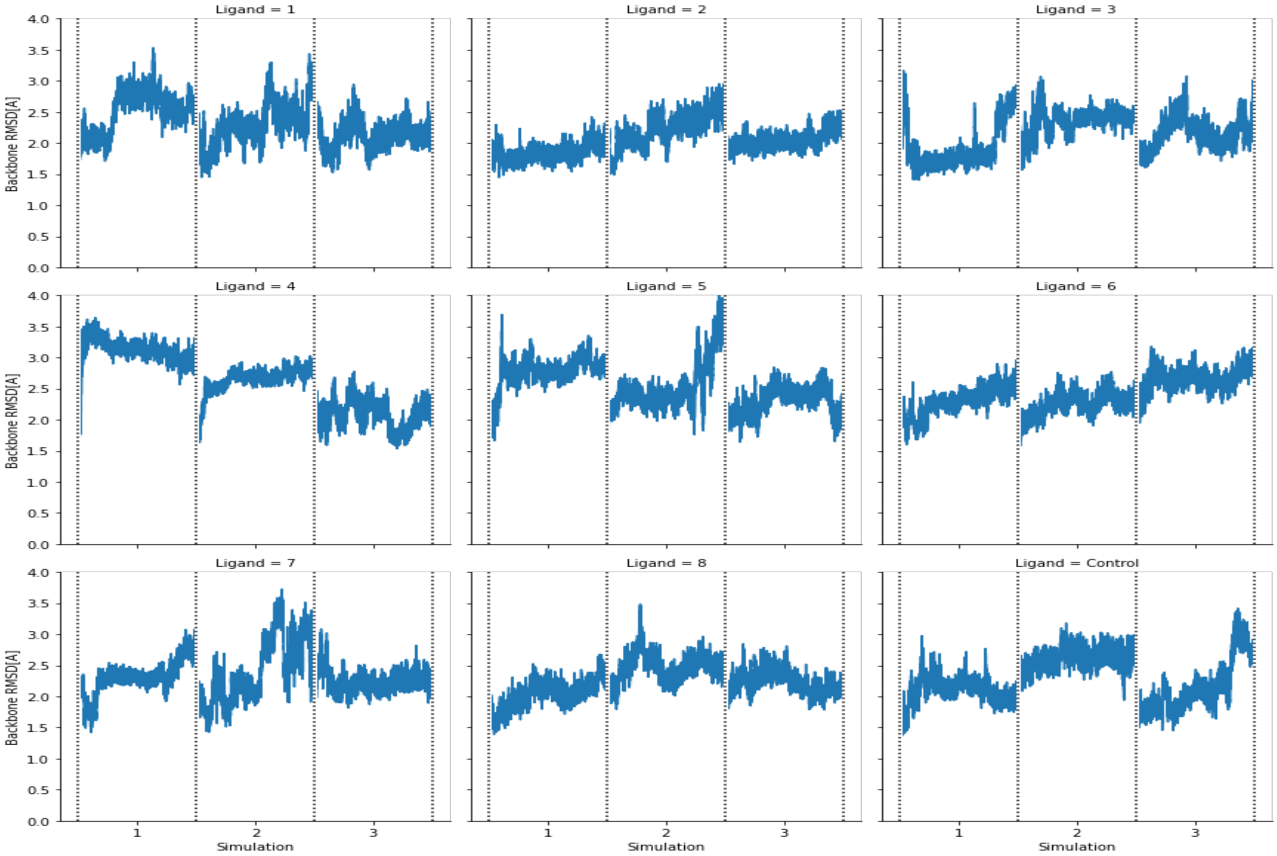

Figure S14. RMSD of CA atoms of the protein during 100 ns simulations repeated 3 times (10 ns truncated as an equilibration period). Non-bound protein used as a control.

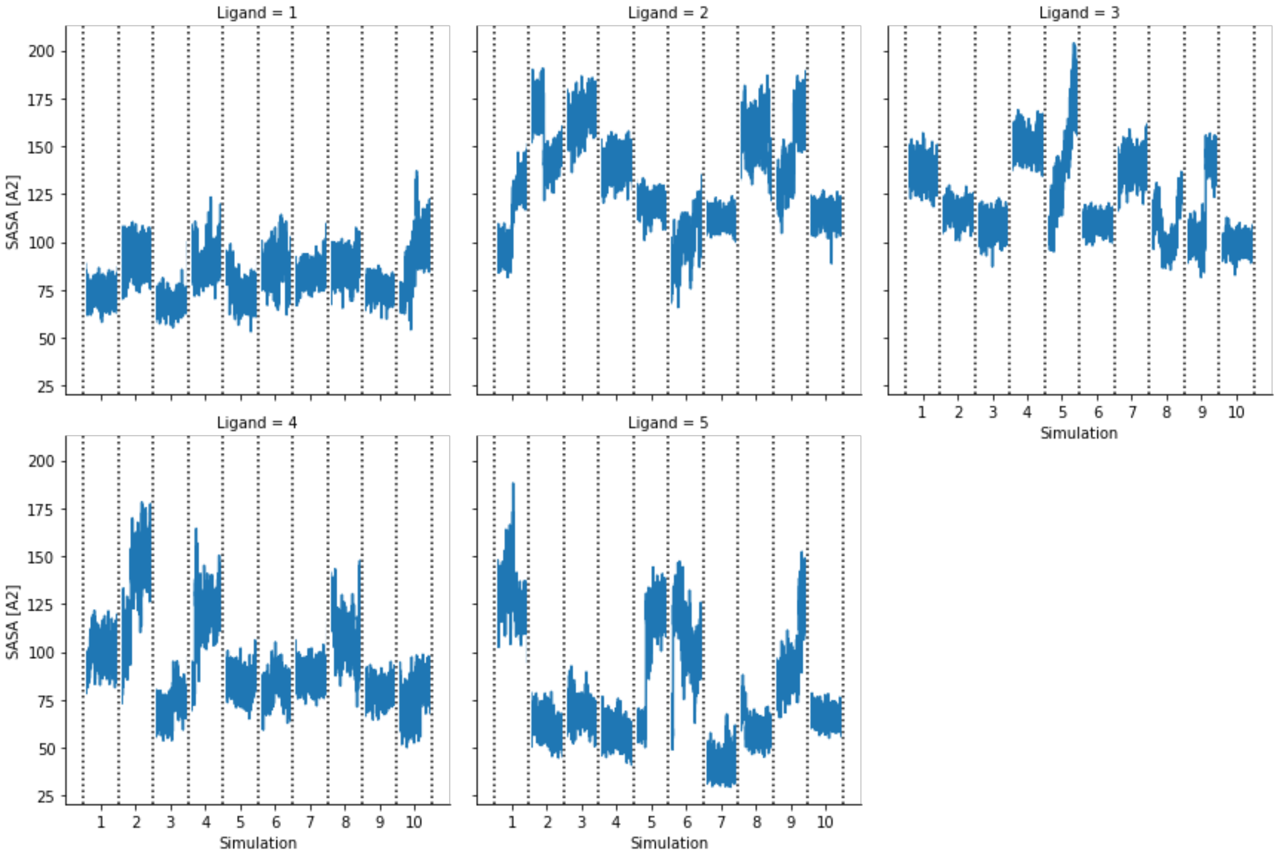

Figure S15. Solvent-accessible surface area of ligands during 20 ns long simulations repeated 10 times (10 ns truncated as an equilibration period).

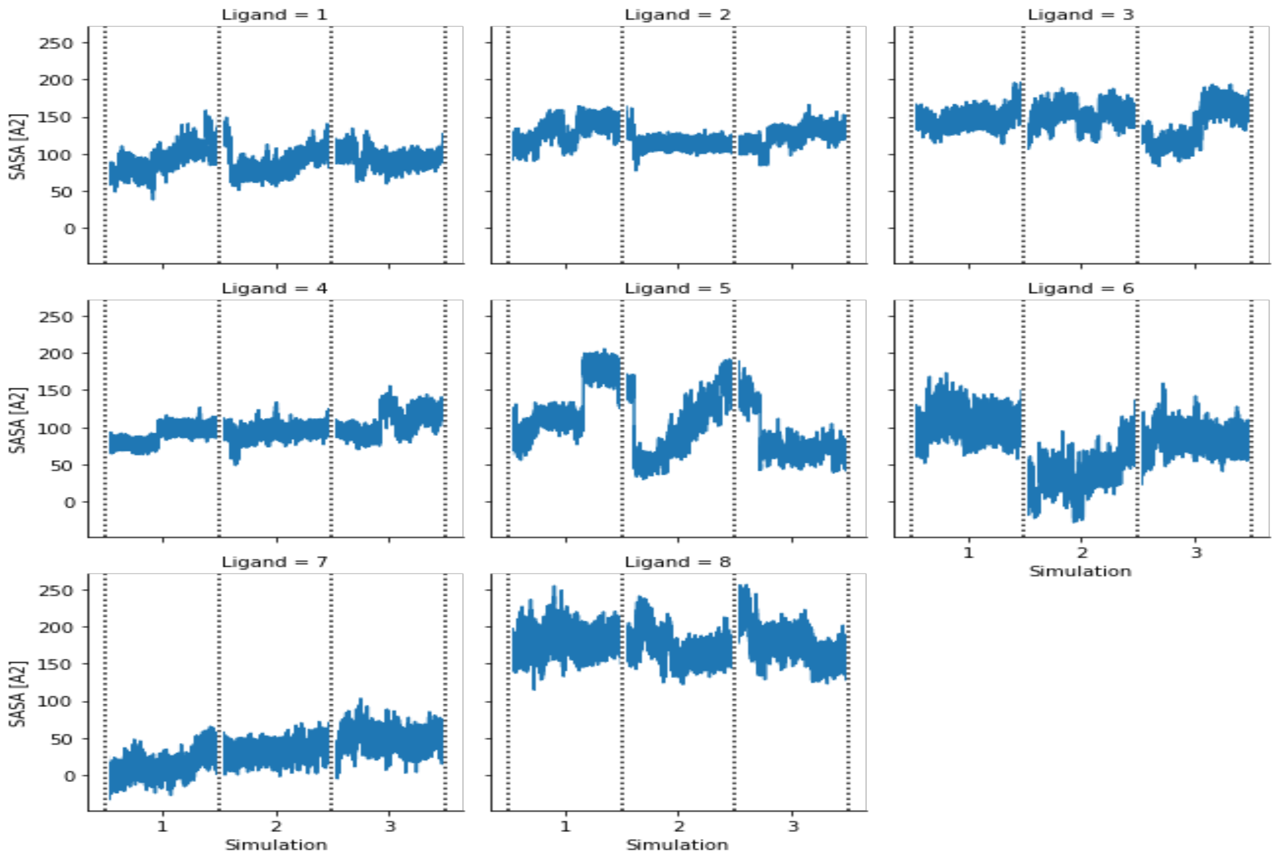

Figure S16. Solvent-accessible surface area of ligands during 100 ns simulations repeated 3 times (10 ns truncated as an equilibration period).

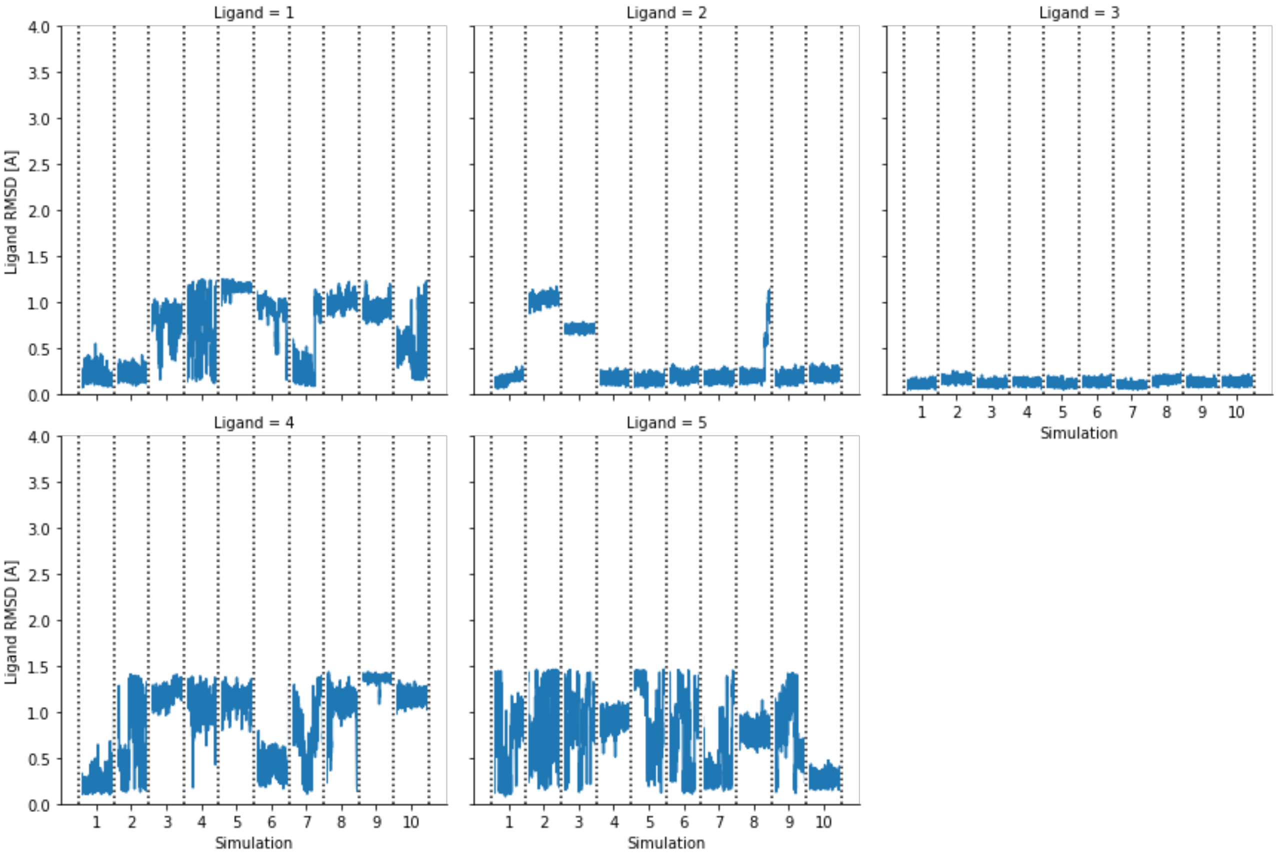

Figure S17. Ligand RMSD during 20 ns long simulations repeated 10 times (10 ns truncated as an equilibration period).

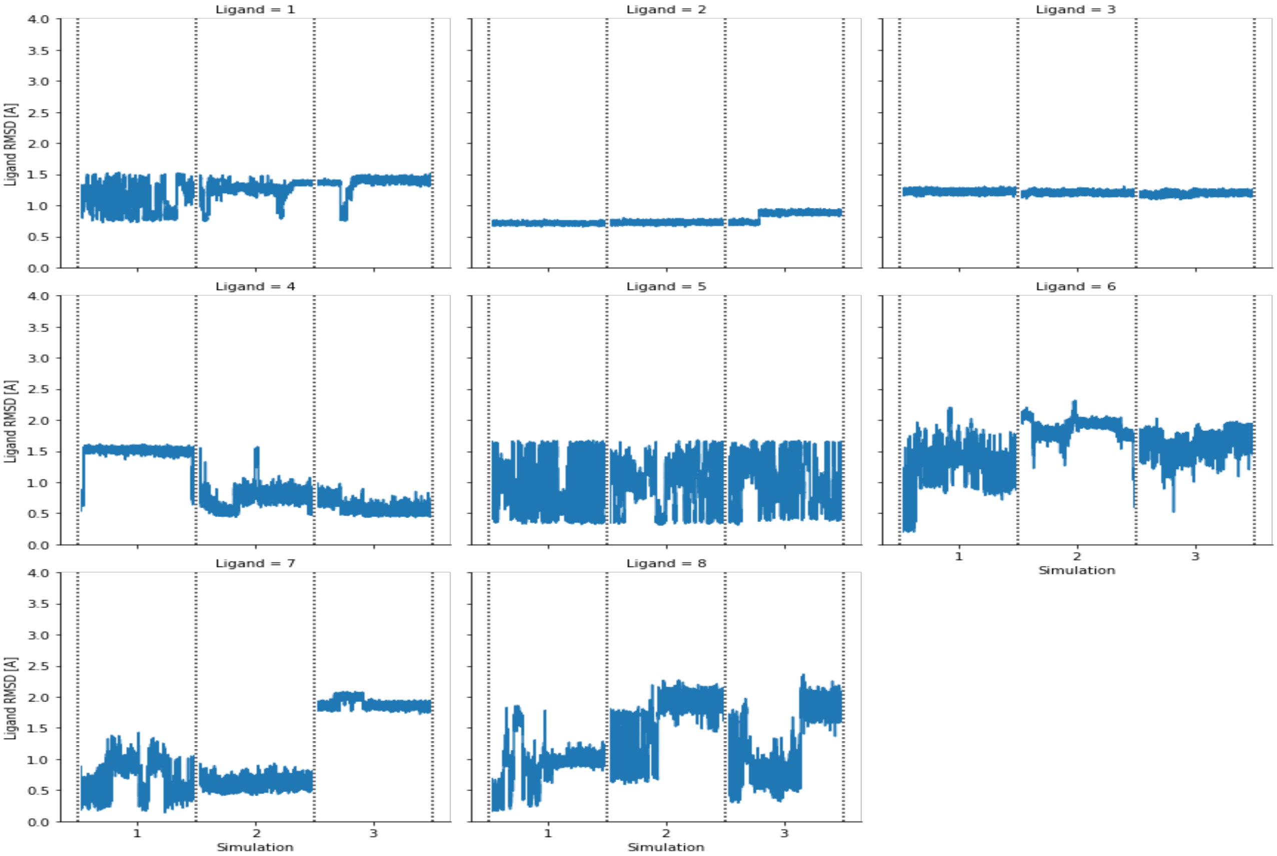

Figure S18. Ligand RMSD during 100 ns simulations repeated 3 times (10 ns truncated as an equilibration period).

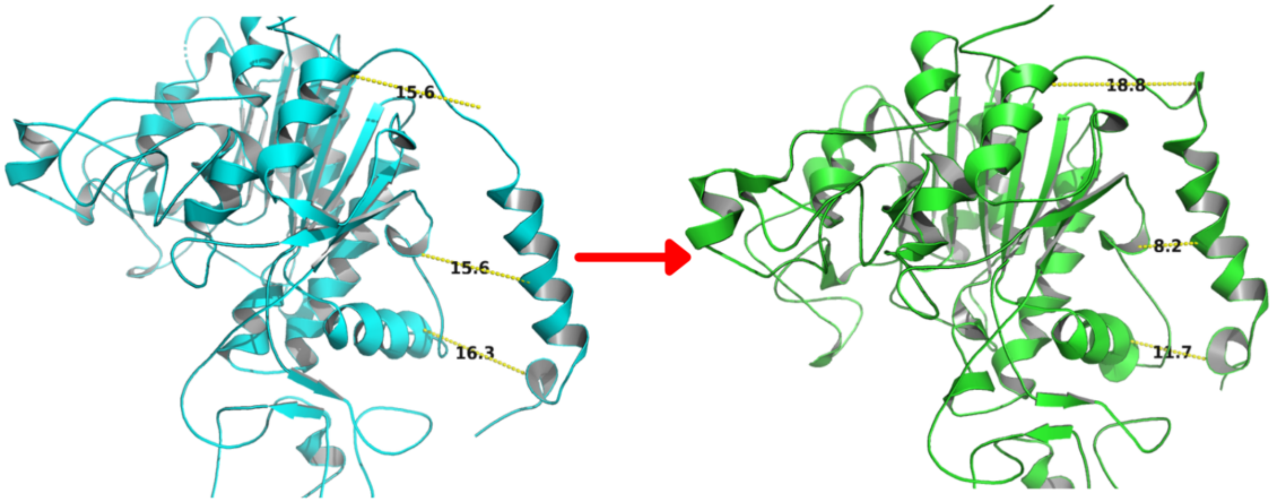

Figure S19. Differences in a terminal α-helix and protein core distances between initial structure and clustered trajectory. Distances are between residues: 6 and 58, 16 and 73, 27 and 470.
